# Does socioeconomic inequality exist in minimum acceptable diet intake among children aged 6–23 months in sub-Saharan Africa? Evidence from 33 sub-Saharan African countries’ demographic and health surveys from 2010 to 2020

**DOI:** 10.1186/s40795-022-00521-y

**Published:** 2022-04-07

**Authors:** Daniel Gashaneh Belay, Asefa Adimasu Taddese, Kassahun Alemu Gelaye

**Affiliations:** 1 Department of Human Anatomy, College of Medicine and Health Sciences, University of Gondar, Gondar, Ethiopia; 2 Department of Epidemiology and Biostatistics, Institute of Public Health, College of Medicine and Health Sciences, University of Gondar, Gondar, Ethiopia

**Keywords:** Minimum acceptable diet, Socioeconomic inequalities, And sub-saharan African

## Abstract

**Background:**

Child undernutrition is a major public health problem in many resource-poor communities in the world. More than two-thirds of malnutrition-related child deaths are associated with inappropriate feeding practices during the first 2 years of life. Socioeconomic inequalities are one of the most immediate determinants. Though sub-Saharan Africa (SSA) shares the huge burden of children undernutrition, as to our search of literature there is limited evidence on the pooled magnitude, socioeconomic inequalities of minimum acceptable diet intake and its contributing factors among children aged 6 to 23 months in the region. This study aimed to assess the level of socio-economic inequalities of minimum acceptable diet intake, and its contributor factors among children aged 6–23 months in SSA using recent 2010–2020 DHS data.

**Methods:**

A total of 78,542 weighted samples from Demographic and Health Survey datasets of SSA countries were used for this study. The data were cleaned using MS excel and extracted and analyzed using STATA V.16 software. The concentration index and curve and wag staff type decomposition analysis were applied to examine wealth-related inequalities in the outcomes. *P*-value < 0.05 was taken to declare statistical significance.

**Results:**

The pooled magnitude of MAD intake among children age 6–23 months in SSA was 9.89% [95%CI: 8.57, 11.21%] ranging from 3.10% in Guinea to 20.40% in Kenya. MAD intake in SSA was disproportionately concentrated on the rich households (pro-rich) [C = 0.191; 95% CI: 0.189, 0.193]. Residence (36.17%), media exposure (23.93%), and women’s education (11.63%) explained the pro-rich inequalities in MAD intake. The model explained 55.55% of the estimated socioeconomic inequality in MAD intake in SSA.

**Conclusion and recommendations:**

Minimum acceptable diet intake in SSA is relatively low. There are moderate socioeconomic inequalities in MAD intake in SSA, mainly explained by residence, media exposure and women’s education. The government of sub-Saharan African countries should plan and work in short terms through the program that endorses women empowerment such as income generation, cash assistance for mothers who have under 2 years of children and women employment using affirmative actions, and nutrition education such as media campaigns and promoting breast feedings. Long-term plans are also needed for those SSA countries with lower income status through programs to enhance their country’s economy to the middle and higher economic level and to improve the wealth index of individual households to narrow the poor-rich gap in the minimum acceptable diet intake.

**Supplementary Information:**

The online version contains supplementary material available at 10.1186/s40795-022-00521-y.

## Background

Nutrition has a big impact on people’s health and is closely tied to social and cognitive development, particularly in children’s formative days [1, 2]. Children cannot receive their complete recommended age-appropriate nutrition in environments with low income and social resources [1]. In children, suboptimal infant and young child feeding (IYCF) practices remain serious public health problems [3]. To overcome these concerns, complementary feeding should be started in children who are 6 months of age and above [2]. Complementary feeding is the introduction of liquids and other foods along with breast milk for 6–23 months age children [4]. World Health Organization (WHO) defines minimum acceptable diet (MAD) practices for 6–23 months age children as a combination of both minimum meal frequency and minimum dietary diversity in both breastfeeding and non-breastfeeding children [5, 6].

In many countries, less than one-quarter of children are reported not getting the nutrition they need to grow well, particularly in the crucial first 1000 days [7, 8]. Child undernutrition is a major public health problem in many resource-poor communities in the world [3]. Among children aged 6 to 23 months from low socioeconomic status, only one in five can feed the minimum recommended diverse diet which is one component of MAD [8].

The first 2 years of age of the child’s life provide an opportunity to ensure the growth, development and survival of the child, through optimum infant and young child feeding (IYCF) practices [4]. Therefore, inappropriate IYCF practices during this period result in significant threats to child health by compromised educational achievement, impaired cognitive development, and low economic productivity which become difficult to reverse later in life [4, 9, 10]. Inappropriate feeding practices during the first 2 years of life are a cause for more than two-thirds of malnutrition-related child deaths [11]. Malnutrition is linked to just half of all deaths of under five children in each year [4, 8]. Optimal complementary feeding practices prevent approximately one-third of child mortality [12]. Research has shown that in sub-Saharan Africa children lost up to 2.5 years of schooling if there was a famine while they were in utero and during their childhood [8].

Even though the minimum acceptable diet problem has multiple causes, it is widely agreed that inadequate IYCF due to socioeconomic inequalities is one of the most immediate determinants [1, 13]. Socio-economic inequalities in child nutrition are a concern for health differences since it is resulting from factors considered to be both avoidable and unfair [14]. The global burden of childhood undernutrition is concentrated in low-income and lower-middle-income countries and becomes a vicious cycle with their economic status [15]. In countries with low socio-economic status with inadequate food and resources, children can not have full growth and developmental possibilities [1]. Sub-Saharan Africa is loaded with half children living in extreme poverty among 385 million around the world, whereas over a third live in South Asia [8]. According to the Global nutrition report in 2020, there were inequalities in dietary diversity, meal frequency, and minimum acceptable diet. Children from the richest households do far better, as do a more educated mother or those who live in urban areas [15, 16]. There were an 11.5% wealth gap, 4.9%, location gap and a 7.7% education gap of minimum acceptable diet intake [16].

A lot of interventions have been taken to overcome these problems [16, 17]. The United Nations (UN) Secretary-General, launched the zero hunger challenge in children, by fulfilling objectives such as; 100% access to adequate food all year round, zero stunted children under 2 years, and sustainability of all food systems [17]. World health organization (WHO) set as strategies for complementary feeding practice by using multiple micronutrient powders for home fortification of foods, and vitamin A for children 6–23 months of age [18]. Despite these lots of interventions that have been taken, the minimum acceptable diet usage is still low [8]. Therefore identifying and reducing the avoidable socioeconomic inequalities of minimum acceptable diet intake and its contributing factors are an important issue in improving the overall health and well-being of the child [14].

There have been studies reporting the burden and determining factors of childhood MAD usage in different countries of sub-Saharan Africa. But those studies were used regionally varied local food items to assess MAD intake among children, which makes it difficult to make pooled estimates and regional comparisons. However, this study used the most recent standard DHS dataset which was collected in a similar design and standardized parameters, makes easy to have pooled prevalence of MAD intake among children.

Therefore this study aimed to assess the pooled prevalence, the level of socio-economic inequalities of MAD intake, and contributor factors for the inequalities among children aged 6–23 months in SSA countries. It will be the crucial point for policymakers to know child nutrition status in the region and draft child nutrition policy and take actions based on the evidence.

## Methods

### Study design, setting, and period

The data source for this study was the recent standard DHS data of Sub-Saharan African countries conducted within 10 years (2010–2020), which was a crossectional study conducted every five-year interval to generate updated health and health-related indicators.

The sub-Saharan is the area in the continent of Africa that lies south of the Sahara and consists of four geographically distinct regions namely Eastern Africa, Central Africa, Western Africa and Southern Africa [19]. But economically, according to the 2019 World Bank list of economies classification categorized as low income (Burundi, Comoros, Ethiopia, Malawi, Mozambique, Rwanda, Tanzania, Uganda, Zambia, Zimbabwe, Cameroon, Chad, the Democratic Republic of the Congo, Gabon, Benin, Burkina Faso, Gambia, Guinea, Liberia, Mali, Niger, Senegal, Sierra Leone, Togo), lower middle income (Kenya, Congo, Zambia, Ivory Coast, Ghana, Lesotho, and Nigeria), and upper-middle-income country (Angola, Namibia, and South Africa) [20]. Together they have a total population of 1.1 billion inhabitants [21]. The datasets are publicly available from the DHS website www.dhsprogram.com [19]. DHS collects data that are comparable across countries. The surveys are nationally representative of each country and population-based with large sample sizes. All surveys use a multi-stage cluster sampling method [22].

### Population

The source population was all children aged 6–23 months preceding 5 years of the survey period across 33 Sub-Saharan African countries. Whereas the study population was children aged 6–23 months preceding 5 years the survey period in the selected Enumeration Areas (EAs) and the mother or the caregiver was interviewed for the survey in each country. Mothers who had more than one child within the 2 years preceding the survey were asked questions about the most recent child [23].

### Sampling procedures and sample size

A total of 47 countries are located in sub-Saharan Africa. Of these countries, only 41 countries had Demographic and Health Survey Report. From these, five countries that did not have a survey report after the 2010/2011 survey year were excluded. These countries are Central Africa Republic (DHS report 1994/95), Eswatini (DHS report 2006/07), Sao Tome Principe (DHS report 2008/09), Madagascar (DHS report 2008/09), and Sudan (DHS report 1989–90). As well as three sub-Saharan Countries (Botswana, Mauritania, and Eritrea) were excluded due to the dataset not being publicly available. Then, after excluding countries that had no DHS report after 2010 and countries where the DHS dataset was not publicly available, a total of 33 countries were included in this study.

Typically, DHS samples are stratified by geographic region and by urban/rural areas within each region. DHS sample designs are usually two-stage probability samples drawn from an existing sample frame. Enumeration Areas (EAs) were the sampling units for the first stage of sampling. In selected EAs, households (HHs) comprise the second stage of sampling. Following the listing of the households, a fixed number of households is selected by equal probability systematic sampling in the selected cluster [22]. The detailed sampling procedure was available in each DHS reports from the Measure DHS website (www.dhsprogram.com) [22].

Weighted values were used to restore the representativeness of the sample data and were calculated from children’s records or kid’s records (KR) DHS datasets. Finally, a total weighted sample of 78, 542 children in the age category of 6–23 months from all 33 countries were included in this study [Table 1].

**Table 1:** Sample size determination of MAD intake and factor associated with it among children age 6–23 months in each sub-Saharan Africa: based on 2010–2020 DHS

Sub-Saharan Africa Countries with Recent DHS reports from 2010/11 to 2019/20				
**Income status**	**Country**	**DHS year**	**Sample size**	
			**Un weighted**	**Weighted**
Lower-income	Burundi	2016/17	4016	4145
	Comoros	2012	727	728
	Ethiopia	2016	2850	3032
	Malawi	2015/16	4642	4664
	Mozambique	2011	3158	3312
	Rwanda	2014/15	1133	1159
	Tanzania	2015/16	3159	3105
	Uganda	2016	4391	4327
	Zimbabwe	2015	1545	1599
	Cameroon	2018	2673	2771
	Chad	2014/15	2791	2878
	DR Congo	2013/14	2572	2495
	Gabon	2012	1112	875
	Benin	2017/18	3965	3968
	Burkina Faso	2010	2080	2099
	The Gambia	2013	1160	1134
	Guinea	2018	1917	1867
	Liberia	2019/20	1538	1359
	Mali	2018	2751	2901
	Niger	2012	1523	1588
	Senegal	2010/11	1349	1262
	Sierra Leone	2019	2685	2669
	Togo	2013/14	1063	1037
	**Subtotal**		**35, 115**	**34,829**
Lower middle income	Kenya	2014	2822	2610
	Congo	2011/12	1504	1339
	Zambia	2018	2851	2780
	Ivory Coast	2011/12	1095	1090
	Ghana	2014	879	864
	Lesotho	2014	468	464
	Nigeria	2018	9211	9292
	Subtotal		**18, 830**	**18, 439**
Upper middle income	Angola	2015/16	4020	3706
	Namibia	2013	644	596
	South Africa	2016	843	827
	**Subtotal**		**5, 507**	**5, 129**
Total			**79,147**	**78, 542**

### Study variables

#### Dependent variables

The outcome variable of this study was taking minimum acceptable diet (MAD) of children 6–23 months which is combined from children who had minimum meal frequency and minimum dietary diversity in both breastfeeding and non-breastfeeding children. During the survey, their mother was asked questions about the types and frequency of food the child had consumed during the day or night before the interview [22].

If a child is taken four out of seven food groups fed during the day or night preceding the survey the following food items are considered as getting minimum dietary diversity. These are Grains, roots and tubers, legumes and nuts, Dairy products (milk, yogurt, and cheese), Flesh foods (meat, fish, poultry, and liver/organ meats), Eggs, Vitamin A-rich fruits and vegetables, and other fruits and vegetables. Whereas minimum meal frequency is the provision of two or more feedings of solid, semi-solid, or soft food for 6–8 months, three or more feedings for 9–23 months breastfeed, and four times for non-breastfed children. The data of the above variables were collected similarly across all SSA countries [6, 22].. Since minimum meal frequency has a different cut-off value for different age groups and breastfed and non-breast feed children, so as the overall meal frequency computed after computing for each group.

#### Independent variables

Socio-demographic factors such as; marital status and household family size, Socioeconomic factors such as; educational attainment of women, occupation of women, and country income status, health behavior factors such as media exposure and breastfeeding status and geographical factors such as place of residence and sub-region in SSA are all taken into account.

The countries income status was categorized as low income, lower middle income, and upper-middle-income country based on the World Bank List of Economies classification since 2019 [20]. World Bank calculated country income based on Gross National Income (GNI) per capita, which categorized as low income $1025 or less; lower middle income, $1026-3995, upper middle income $3996-12,375,and high income $12,375 or more [20].

### Data processing and analysis

This study was performed based on the DHS data obtained from the official DHS measure website www.measuredhs.comafter permission has been obtained via an online request by specifying my objectives. Data from the DHS dataset were downloaded standard DHS data in STATA format then cleaned, integrated, transformed, and append to produce favorable variables for the analysis. Microsoft Excel and STATA 16 software were used to generate both descriptive and analytic statistics of the appended 33 countries’ data to describe variables in the study using statistical measurements. The variance inflation factor (VIF) was used to detect multicollinearity, and all variables had VIF values less than 10 and the mean VIF value of the final model was 1.57.

The pooled estimate of MAD intake among children in Sub- Saharan Africa and Sub-regions was estimated using the metan STATA command.

### Model building

#### Concentration curve and index

The concentration index and concentration graph approach are used to examine socioeconomic inequalities in health outcomes [24, 25]. The concentration curve is used to identify whether socioeconomic inequality in some health variables exists and whether it is more pronounced at one point. It displays the share of health accounted for by cumulative proportions of individuals in the population ranked from the poorest to the richest [25, 26].

The two key variables underlying the concentration curve are the health variable and the distribution of the subject of interest against the distribution of the variable capturing living standards [27]. The concentration curve plots the cumulative percentage of MAD usage (y-axis) against the cumulative percentage of children 6–23 months ranked by living standards beginning with the poorest and ending with the richest (x-axis) households [27].

A 45^0^ line running from the bottom left-hand corner to the top right-hand corner concentration curve would be indicated the absence of inequity. Furthermore, the concentration curve lying above the equality line (45^0^) indicated that MAD intake is disproportionately concentrated between poor and whereas below the equality line (45^0^) indicated concentrated on rich [28].

To quantify and compare the degree of socio-economic related inequality in MAD intake, concentration index (C) is used [26, 29] and it is twice the area between the concentration curve and the line of equity with the range of − 1 to + 1. The sign indicates the direction of the relationship between MAD intake and the distribution of living standards (wealth status) [25, 27].

$$C=\frac{2}{n\mu}\sum_{i=1}^n hiRi-1$$

Where hi is the health outcome (MAD intake in this study), μ is the mean of *hi* and *n* is the number of people. Ri represents the fractional rank of individual i in the living standards distribution used (the wealth index), with i taking the value of 1 for the poorest and the value of n for the richest [27, 29, 30].

As a result, C > 0 showed that MAD intake was disproportionately concentrated on the rich (pro-rich), and CI < 0 revealed that the MAD intake is disproportionately concentrated on the poor (pro-poor) [27, 28] whereas C = 0 indicated that the distribution is proportionate. Accordingly, C = 1 showed that the richest person had children taken MAD, whereas C = − 1 indicated that the poorest person had all of the children taken MAD [27, 30].

But the outcome variable in the present study is binary (taken/not taken MAD), the bounds of C depend on the mean (μ) of the outcome variable and do not vary between 1 and − 1. Thus the bounds of C vary between μ–1 (lower bound) and 1–μ (upper bound) and the interval shrink when the mean (μ) increases. As a correction, the present study applied the Wag staff normalization to calculate the concentration index by dividing C by 1 minus the mean (1–μ) [27, 30].

$${\mathrm{C}}_{\mathrm{Normalized}}=\frac{c}{1-\mu }$$

#### Wag staff decomposition analysis

Wag staff-type decomposition analysis was performed for those variables that were screened statistical significance based on multi-level analysis and clinical significance after the concentration index and curve were assessed and showed income-related inequality to the magnitude of MAD usage. The Wag staff-type decomposition analysis quantifies the degree of income-related inequalities of the minimum acceptable diet intake and explains the contribution of each factor to the observed inequality [31]. Concentration index (C) decomposed based on regression analysis of the relationship between an outcome variable and a set of determinants. The overall concentration index can be decomposed into k social determinant contributions, in which each social determinant’s contribution is obtained by multiplying the sensitivity of the outcome (MAD) related to that determinant and the degree of income-related inequality in that factor [27, 32].

Based on a linear additive regression model, the concentration index for minimum acceptable diet intake (y) can be expressed as follows.

$$C=\sum k\left(\beta k\overline xk/\mu\right)\;Ck+GC_\varepsilon/\mu$$

Where μ is the mean of y *x* ® *k* is the mean of Xu, Ck is the concentration index of xk, and GCε (residual) is the generalized concentration index for the error term (*ε*). The overall concentration index of MAD intake (y) includes the explained part which is the sum of the contributions of k determinants, and the unexplained part (residual). Based on the Wag staff normalization, the normalized decomposition of the concentration index, obtained by dividing the concentration index by 1–μ [30]. Absolute contribution is expressed in the same unit as the C whereas relative contribution was the percentage of the C of each covariate to the total observed income-related inequality in MAD.

### Data quality control

The DHS data are comparable across countries. The missing values were clearly defined by the DHS guideline. If there were missing values and “don’t know” in breastfeeding, assumed as not breastfeeding, but if there were in specific foods, excluded from further analysis [22]. The magnitude of MAD usage among children in each country was compared with the respective DHS reports.

## Results

### Socio demographic characteristics of mothers or caregivers

A total weighted sample of 78,542 children of age 6–23 was included in this study. Almost equal proportions of males (50.49%) and females (49.51%) were studied. Nearly two-thirds (65.42%) of the children were found in the age group from 12 to 23 months with a median age of 14 (IQR: 8) months. Four-fifths (79.49%) of the children were breastfeeding. Three-fourths (75.52%) of mothers of children were in the age group of 20–35 years, with a median age of 27 (IQR: 16) years. More than three-fifths of women (61.7%) had formal education. Most (70%) of the mothers were not working. Most (70%) of the respondents were rural inhabitants. Sixty-four percent of the Sub-Saharan African countries included in the study were lower-income countries [Table 2].

**Table 2:** Socio-demographic characteristics of the study mothers/caregivers in a study of MAD intake and associated factors among children age 6–23 months in Sub-Saharan Africa: based on 2010–2020 DHS

Variables	Categories	Frequency (n)		Weighted percentage(%)
		Unweighted	Weighted	
Sex of child	Male	40,056	39,657	50.49
	Female	39,091	38,885	49.51
Age of child	6–8 months	14,301	14,097	17.95
	9–11 months	13,035	13,066	16.64
	12–23 months	51,811	51,378	65.42
Age of women (years)	15–19	7500	7305	9.30
	20–35	59,774	59,541	75.81
	36–49	11,873	11,696	14.89
Breast feeding status	Not breastfed	16,232	16,039	20.51
	Breastfed	62,915	62,503	79.49
Educational attainment of women	No education	30,314	29,673	37.78
	Prim. education	27,104	26,888	34.23
	Sec. & above	21,729	21,981	27.99
Occupation of women	Worked	23,008	22,214	29.44
	Not working	53,010	53,239	70.56
Marial status of mother	Merried	55,160	55,286	70.39
	Not merried	23,987	23,255	29.61
House hold family size	1–4	21,823	22,124	28.17
	5–10	47,473	46,784	59.57
	> 11	9851	9633	12.26
Media exposure	No	30,381	29,187	37.21
	Yes	48,670	49,261	62.79
Wealth index	Poorest	19,951	17,993	25.21
	Poorer	17,558	17,200	22.18
	Midddle	15,790	15,958	19.95
	Richer	13,803	14,634	17.44
	Richest	12,045	12,757	15.22
Country income level	Lower	51,015	51,329	64.46
	Lower middle	21,503	21,209	27.17
	Upper middle	6629	6003	8.38
Residence	Urban	23,782	24,533	31.24
	Rural	55,365	54,009	68.76
Region in SSA	Centeral Africa	14,682	14,064	17.91
	East Africa	31,294	31,462	40.06
	West Africa	31,216	31,130	39.64
	South Africa	1955	1886	2.4

### The pooled magnitude of minimum acceptable diet intake among children aged 6–23 months

**Fig. 1:**
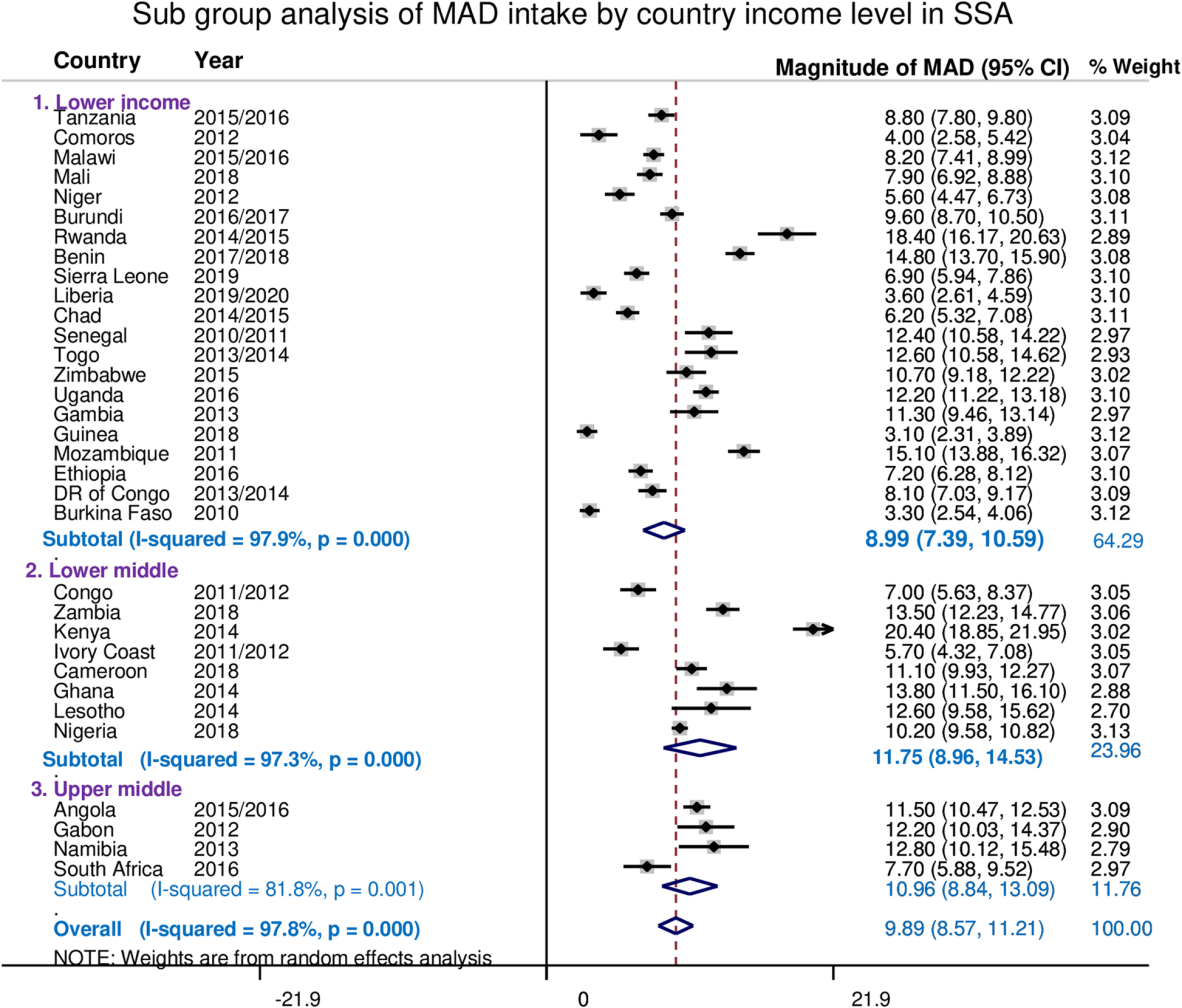
The Forest plot showed that pooled magnitude of MAD intake among 6–23 children in SSA based on income status

The overall pooled estimate of the minimum acceptable diet intake among children aged 6–23 months in Sub-saharan African countries was 9.87% (95%CI: 8.57, 11.21%), with I^2^ = 97.8% and ranging from 3.10% in Guinea to 20.40% in Kenya. Moreover, the pooled magnitude of MAD intake across country income levels was determined. The pooled estimate of MAD intake in low-income countries was 8.99% (95%CI: 7.39, 10.59%), lower-middle-income countries 11.75%(95%CI: 8.96, 14.53%), and 10.96% across upper middle-income countries (95%CI: 8.84, 13.04%) (Fig. 1).

### Wealth related inequality in minimum acceptable diet usage

#### Concentration index and curve

The concentration index is used to quantify the degree and show the direction of socio-economic-related inequality in a health variable. The value of negative sign indicates the more concentration of MAD intake among the poor, where a positive value indicates concentration among the rich.

**Fig. 2:**
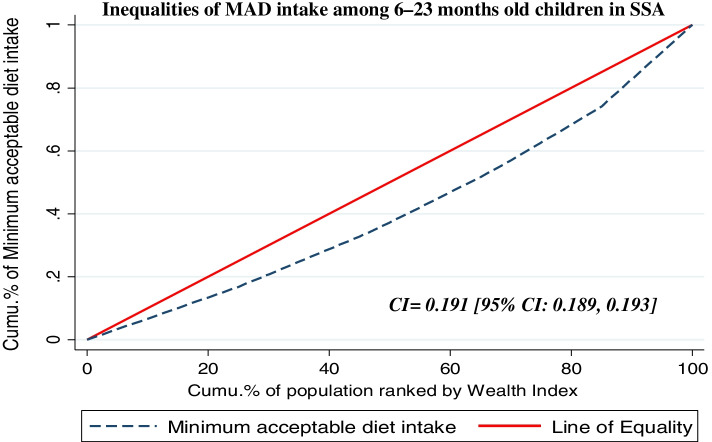
Wealth-related inequality of minimum acceptable diet intake among children age 6–23 months in Sub-Saharan Africa

In this study, the overall wag staff normalized concentration index (C) analyses of the wealth-related inequality of MAD showed that the pro-rich distribution of MAD intake with [C = 0.191; 95% CI: 0.189, 0.193]. This shows that MAD intake among children aged 6–23 months was disproportionately concentrated on the richer groups (pro-rich). The concentration index is twice the area between the concentration curve and the diagonal line (Fig. 2). Then when multiplying the C by 75 (0.191*75) =14.33, which showed that 14.33% of the MAD intake would need to be (linearly) redistributed from the richer half to the poorer half of the population to arrive at a distribution with an index value of zero (perfect equality). The finding from the indices is in agreement with the results of the concentration curves in Fig. 2. Similarly, the concentration curve in the following figures showed that the concentration graph of minimum acceptable diet usage was below the line of equality which indicated that the distribution of minimum acceptable diet used children was concentrated in rich households (pro-rich distribution) [Fig. 2].

**Fig. 3:**
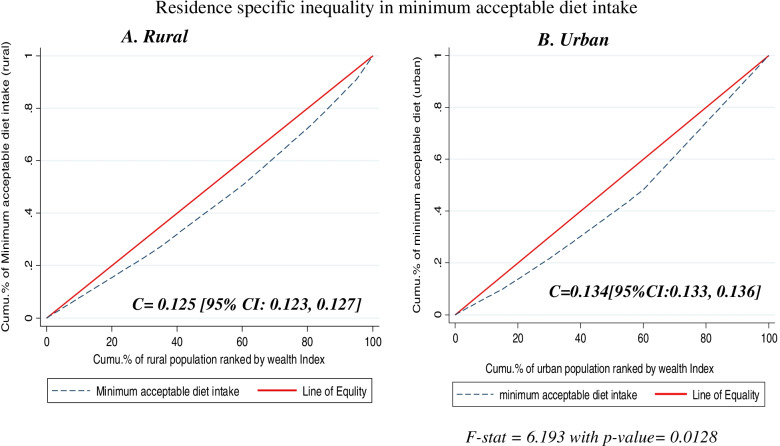
Residence-specific wealth-related inequality in minimum acceptable diet intake among children age 6–23 months in sub-Saharan Africa

The wealth-related inequality of MAD intake was significantly higher among the urban (0.134) residents than rural (0.125) (*p*-value = 0.013) and similarly, the concentration curve showed that the concentration graph of MAD intake among children aged 6–23 months who were live in urban residence was below the graph of rural residence (Fig. 3).

### The wag staff decomposition analysis

After the concentration index and curve were assessed and showed income-related inequality to the MAD intake, wag staff-type decomposition analysis have been fitted for those variables that were statistically significant during multi-level analysis and clinical important variables for wealth-related changes. The wag staff-type decomposition analysis is used to decompose the overall income-related inequalities of the MAD intake by variables and explains the contribution of each factor to the observed inequality.

Table 3 reveals the wag staff decomposition analysis for the contribution of the various explanatory variable for wealth inequalities of MAD intake among children aged 6–23 months in sub-Saharan African countries. The table contains information about coefficient, elasticity, Concentration(C), absolute contribution, and percent contribution. Elasticity is the sensitivity of MAD intake for each factor. The concentration index in each variable is the degree and direction of socio-economic-related inequality in MAD intake corresponding to specific explanatory variables. The value of negative sign in C indicates the more concentration of MAD intake among the poor, where a positive value indicates concentration among the rich. Absolute contribution is calculated by multiplying elasticity with the concentration index of each factor and indicates the extent of inequality contributed by the explanatory variables. Whereas percent contribution means the contribution of each variable to the overall concentration index.

**Table 3:** Decomposition of the concentration index of wealth-related inequalities for MAD intake status among children aged 6–23 months in SSA evidence by 2010 to 2020 DHS

Variables	Categories	Coeffecient [95% CI]	Elasticity	C	Cont.^a^	%Cont.^b^
Socio demographic factors (subtotal)					**0.000**	**−0.12**
Marital status of the mother	Married (Ref.)	–	–	–	–	–
	Not merried	−0.019[− 0.024, 0.015]*	−0.029	0.006	0.000	− 0.10
Household family size	1–4 (Ref.)	–	–	–	–	–
	5–10	0.003[− 0.002, 0.007]	0.009	−0.028	0.000	−0.13
	> 11	0.001[−0.004, 0.005]	0.008	0.007	0.000	0.03
Socio-economic factors (subtotal)					**0.016**	**8.25**
Educational attainment of women	No education (Ref.)	–	–	–	–	–
	Primery education	0.011[0.005, 0.017]**	0.017	−0.098	− 0.002	−0.85
	Secondary & above	0.046[0.039, 0.053]**	0.046	0.483	0.022	11.63
Occupation of women	Not worked (Ref.)	–	–	–	–	–
	Worked	0.017[0.012, 0.021]**	0.059	−0.077	− 0.004	−2.34
Country income status	Low (Ref.)	–	–	–	–	–
	Lower middle	0.001[0.005, 0.015]**	0.023	−0.001	0.000	−0.02
	Upper middle	0.015 [0.007, 0.026]*	0.010	−0.030	0.000	−0.16
Health behavior factors (subtotal)					**0.023**	**11.94**
Media exposure	No (Ref.)	–	–	–	–	–
	Yes	0.034[0.030, 0.039]**	0.108	0.425	0.046	23.93
Breastfeeding	No (Ref.)	–	–	–	–	–
	Yes	0.037[0.033, 0.041]**	0.140	−0.164	−0.023	−11.9
Geographical factors (subtotal)					**0.068**	**35.58**
Residence	Urban (Ref.)	–	–	–	–	–
	Rural	−0.037[− 0.043, − 0.032]*	−0.105	− 0.659	0.069	36.12
Region in SSA	Central Africa (Ref.)	–	–	–	–	–
	East Africa	0.031[0.023, 0.038]***	0.057	−0.017	−0.001	− 0.51
	West Africa	−0.001[− 0.007, 0.007]	−0.003	0.030	0.000	−0.04
	South Africa	0.009 [−0.007, 0.025]	−0.015	− 0.029	0.000	0.02
	Explained inequality				0.106	**55.55**
	Residual				0.085	44.42
	Overall Inequality				0.191	100.00
	95% conf. Interval			0.189, 0.193		

* = *P*-value < 0.05, ** = *P*-value < 0.01, *** = *P*-value < 0.001

Ref. = Reference catagory; C = concentration index

^a^ Cont. C = Contrburion to concentration index = C *Elasticity

^b^ %Cont = Percentage contrbution to concentration index = (Cont.C/ Over all conc. index)*100

In this study, more than half (55.55%) of the wealth-related inequalities of MAD intake in children were explained by the combination of variables fitted in the model.

Geographical-related factors contribute most of (35.58%) the pro-rich wealth-related inequality on the usage of MAD among children. More than one-third (36.12%) of the pro-rich inequalities in MAD taking among children is explained by the residents. Having media exposure also explained nearly one-fourth (23.93%) of the pro-rich wealth-related inequality for children who had taken MAD. The other 11.63% of the estimated pro-rich inequalities in MAD usage are explained by maternal secondary educational status [Table 3].

## Discussions

Inadequate infant and young child feeding (IYCF) practices are the major determinants of undernutrition, optimal growth, and development, especially in the first 2 years of life is a major problem both globally and in developing countries [33]. Identifying and reducing avoidable socioeconomic inequalities and other determinants of malnutrition is a critical step toward improving children’s overall health and well-being [14]. This study aimed to determine the pooled estimate, socio-economic inequalities of minimum acceptable diet intake, and contributor factors among children less than 2 years in sub-Saharan Africa. Based on this, only 9.89% [95% CI: 8.57, 11.21%] of children aged 6–23 months in Sub-Saharan African countries can access a minimum acceptable diet and MAD intake among children age 6–23 mo in SSA was disproportionately concentrated on the rich (pro-rich) households [C = 0.191; 95% CI: 0.189, 0.193]. This pro-rich inequality in MAD intake was explained by maternal educational status, having media exposure, and residence.

The lowest magnitude (9.89%) of MAD intake in our study is in line with research conducted in India which was 9% [34]. But lower than a multi-site study conducted in America, Asia, and Africa 21% [35], South Asia countries [36], Bangladesh 20% [37], and Indonesia 40% [38] of children aged 6–23 can access a minimum acceptable diet. The discrepancy might be due to geographical variation, population growth, and socio-economic status of the countries [35]. Cultural beliefs and knowledge paradigms about MAD are also known to influence feeding practices [4, 34]. Studies showed that growth faltering among Sub-saharan African children becomes evident from early infancy and is sustained through the second year of life which is the period with the highest reported prevalence of overall malnutrition [39]. But our finding is higher than a study conducted and in the Philippines 6.7% [40] of children aged 6–23 can access a minimum acceptable diet. This is due to the that, the current study included a large population from different geographic Sub-saharan African regions with various cultures, beliefs, and traditions which make it a real estimation of the magnitude in SSA.

In this study, a significant variation of MAD usage of children among SSA countries was observed, in which Kenya (20.40%) had a significantly higher, whereas, Guinea (3.10%) had a statistically significant lowest magnitude of MAD usage am children. This is in line with a study in India [34], Indonesia [3], South Asia [36], and West African countries [41] which reported regional variation in MAD usage. This might relate to the difference in governmental actions toward the application of national nutritional programs, and addressing cultural beliefs around complementary feeding [42]. For instance, the better magnitude of MAD intake in Kenya was achieved by implementing a health platform which is called the Baby-Friendly Community Initiative platform and by integrating WASH (Water, Sanitation and Hygiene) into complementary feeding sessions [42]. The availability and accessibility of foods in the region may have also a contribution. Children in agrarian dominant and city dwellers were more likely to have MAD [3, 43, 44]. For instance. Guinea is among the poorest countries in the world which ranks 179 of 187 countries with 10% population were food insecure [45]. Therfore this low magnitude MAD intake might be associated with it. Evidence also showed that there is an ecological association between dietary diversity and child nutrition in SSA, due to ecology-specific crops production and livestock farming [39].

In this study, we found that the concentration index and curve result showed that the MAD intake was disproportionately concentrated on the rich (pro-rich) households [C = 0.191; 95% CI: 0.189, 0.193]. This is in line with a study conducted in India [46, 47], South Asia [36], Tanzania [48]. It is known that children from a family of higher-income can feed diversified foods and frequently as their families could be more likely to afford to have diversified foods as compared to children from a low household income [49]. In this study, the pro-rich inequalities in MAD intake were explained by maternal educational status, having media exposure of household, and living in a rural residence.

The contribution of secondary and above maternal education towards explaining wealth-related inequality of MAD intake in this study was positive. The result was consistent with studies in India [46, 47, 50]. The study in New York also showed that the association of maternal education and child nutrition was positive in intermediate and high socioeconomic conditions [51]. The global nutrition report 2020 also pointed out that, the education gap contributes 7.7% of child nutrition inequalities [16]. This is might be due to those children of educated mothers having health advantages due to their higher socioeconomic status [47]. Maternal schooling can help to foster the positive association between household wealth and child linear growth [52].

Media usage of the household also has a large contribution to explaining pro-rich wealth-related inequality of MAD intake among children aged 6–23 mo in SSA. This might be due to that those media user households have more likely to be the richest and eventually to feed MAD for their kids.

In this study, the concertation of the rural residence was negative for pro-rich wealth-related inequalities of MAD intake. This is in line with studies conducted in India [47, 53]. According to the global nutrition report 2020, the location gap contributes 4.9% of child nutrition inequalities [16]. This is due to that factors that determine nutritional status differ between urban and rural areas. Nutrition in urban children is characterized by life events of their residence which have a greater dependence on cash income but lower reliance on agriculture and natural resources [54]. It is also supported by the multilevel result of this study which showed rural areas had a lower likelihood for MAD intake and only 4.15% of children from rural areas belonged to the richest household wealth status whereas was two-fifths (40%) of the urban, which resulted in a negative contribution.

The main strength of this study was the use of the weighted nationally representative data of each Sub-saharan African country with a large sample which makes it representative at Sub-Saharan and regional levels. Therefore, it has appropriate statistical power that can be generalized of the estimates in minimum acceptable diet intake in the study setting to all children 6–23 during the study period. Furthermore, the concentration index and curve and wag staff decomposition analysis are appropriate statistical models to shows the direction and degree of socioeconomic inequality of MAD between the poorest to the richest household.

Since the data were collected cross-sectional at a different point in time by self-reported interview would be prone to recall and social desirability bias. The drawback of the secondary nature of data was inevitable. The heterogeneity of the pooled estimate of MAD intake was not managed by further analysis.

## Conclusion and recommendations

The proportion of minimum acceptable diet usage among children aged between 6 and 23 months in Sub-saharan Africa was relatively low.

Minimum acceptable diet intake was disproportionately concentrated on the rich households (pro-rich concentration). Secondary and above maternal education, having media exposure of household and rural residence were positively contributor whereas, breastfeeding was a negative contributor for pro-rich socioeconomic inequalities in MAD intake.

To increase minimum acceptable diet intake among children age 6–23 months in sub-Saharan Africa, policymakers in nutritional projects and other stakeholders should work as an integrated approach with other sectors, and give prior attention to modifiable socio-economic factors such as promoting women’s education and employment, increase wealth status, and media exposure of the household, and promoting breastfeeding behavior.

The government of sub-Saharan African countries should plan and work in short terms through the program that endorses women empowerment such as income generation, cash assistance for mothers who have under 2 years of children and women employment using affirmative actions, and nutrition education such as media campaign and promoting breastfeedings. Long-term plans are also needed for those SSA countries with lower income status through programs to enhance their country’s economy to the middle and higher economic level and to improve the wealth index of individual households. Interventions to improve MAD practice should not only be implemented factors at the individual level but also be tailored to the community context. SSA especially in East Africa regions needs equity-focused interventions to curb the inequalities and low magnitude of MAD intake, not only by taking measures for economic equity but also need the balance by supporting the marginalized group such as uneducated women, households with no media usage, and rural residence.

## Supplementary Information

**Additional file 1.** Ethical clearance from the International Review Board of Demographic and Health Surveys (DHS) program data archivists.

## Data Availability

Data is available publically access from the open databases. It can be accessed by the following website.https://dhsprogram.com/data/dataset_admin/login_main.cfm?CFID=10818526&CFTOKEN=c131014a480fe56-4E0C6B7F-F551-E6B2-50. There are no financial, non-financial, and commercial organizations competing of interests.

## References

[1] Black RE (2013). Maternal and child undernutrition and overweight in low-income and middle-income countries. Lancet.

[2] Organization, W.H. and P.A.H. Organization (2001). Guiding principles for complementary feeding of the breastfed child.

[3] Ng CS, Dibley MJ, Agho KE (2012). Complementary feeding indicators and determinants of poor feeding practices in Indonesia: a secondary analysis of 2007 demographic and health survey data. Public Health Nutr.

[4] Gizaw G, Tesfaye G (2019). Minimum acceptable diet and factor associated with it among infant and young children age 6-23 months in north Shoa, Oromia region, Ethiopia. Int J Homeopathy Nat Med.

[5] IYCF, W.h.o.W (2007). Indicators for assessing infant and young child feeding practice.

[6] (WHO), W.h.o (2010). Nutrition Indicator Reference Sheets: External Source Data.

[7] Nkoka O, Mhone TG, Nanda PA (2018). Factors associated with complementary feeding practices among children aged 6–23 mo in Malawi: an analysis of the demographic and health survey 2015–2016. Int Health.

[8] Unicef (2019). Children, food and nutrition, and growing well in a changing world.

[9] Beyene M, Worku AG, Wassie MM (2015). Dietary diversity, meal frequency and associated factors among infant and young children in Northwest Ethiopia: a cross-sectional study. BMC Public Health.

[10] Molla M, Ejigu T, Nega G. Complementary Feeding Practice and Associated Factors among "Mothers Having Children 6–23 Months of Age, Lasta District, Amhara Region, Northeast Ethiopia", Advances in Public Health. 2017;2017:8. Article ID 4567829. 10.1155/2017/4567829.

[11] Solomon D, Aderaw Z, Tegegne TK (2017). Minimum dietary diversity and associated factors among children aged 6–23 months in Addis Ababa, Ethiopia. Int J Equity Health.

[12] Feleke FW, Mulaw GF (2020). Minimum acceptable diet and its predictors among children aged 6-23 months in Mareka District, southern Ethiopia: community based cross-sectional study. Int J Child Health Nutr.

[13] Ahoya B, Kavli JA (2019). Accelerating progress for complementary feeding in Kenya: Key government actions and the way forward. Matern Child Nutr.

[14] Peeters A, Blake MR (2016). Socioeconomic inequalities in diet quality: from identifying the problem to implementing solutions. Curr Nutr Rep.

[15] Vollmer S (2017). Levels and trends of childhood undernutrition by wealth and education according to a composite index of anthropometric failure: evidence from 146 demographic and health surveys from 39 countries. BMJ Glob Health.

[16] Research DIP (2020). Global nutrition report: action on equity to end malnutrition.

[17] WHO Document Production Services, G., Switzerland Global nutrition policy review: What does it take to scale up nutrition action? 2013.

[18] (WHO), W.H.O., Essential Nutrition Actions: improving maternal, newborn, infant and young child health and nutrition. 2013. 25473713

[19] USAID. DHS program demographic and health surveys. 2020; Available from: https://dhsprogram.com/data/dataset_admin/login_main.cfm?

[20] Bank W (2019). World Bank List of Economies (June 2019)..

[21] Worldometr, Subregions in Africa by population (2021). 2021.

[22] Croft, et al., Guide to DHS Statistics. 2018: Rockville, Maryland, USA: ICF.

[23] The DHS Program ICF Rockville, M., USA, Nigeria demographic and health survey 2018. 2018.

[24] Kakwani N, Wagstaff A, Van Doorslaer E (1997). Socioeconomic inequalities in health: measurement, computation, and statistical inference. J Econ.

[25] Debie A (2020). Complete vaccination service utilization inequalities among children aged 12–23 months in Ethiopia: a multivariate decomposition analyses. Int J Equity Health.

[26] Wagstaff A, Paci P, Van Doorslaer E (1991). On the measurement of inequalities in health. Soc Sci Med.

[27] Wagstaff A, O'Donnell O, Van Doorslaer E, Lindelow M. Analyzing health equity using household survey data: a guide to techniques and their implementation. World Bank Publications; 2007.

[28] Heckley G, Gerdtham U-G, Kjellsson G (2016). A general method for decomposing the causes of socioeconomic inequality in health. J Health Econ.

[29] Wagstaff A, Watanabe N. Socioeconomic inequalities in child malnutrition in the developing world. World Bank Policy Research Working Paper. 2000 Sep 30(2434).

[30] Amroussia N, Gustafsson PE, Mosquera PA (2017). Explaining mental health inequalities in northern Sweden: a decomposition analysis. Glob Health Action.

[31] Saidi O (2019). Explaining income-related inequalities in cardiovascular risk factors in Tunisian adults during the last decade: comparison of sensitivity analysis of logistic regression and Wagstaff decomposition analysis. Int J Equity Health.

[32] Vaezghasemi M (2020). Decomposition of income-related inequality in upper secondary school completion in Sweden by mental health, family conditions and contextual characteristics. SSM-Popul Health.

[33] Marriott BP (2011). World Health Organization (WHO) infant and young child feeding indicators: associations with growth measures in 14 low-income countries.

[34] Dhami MV (2019). Prevalence and factors associated with complementary feeding practices among children aged 6–23 months in India: a regional analysis. BMC Public Health.

[35] Lutter CK (2011). Undernutrition, poor feeding practices, and low coverage of key nutrition interventions.

[36] Senarath U (2012). Comparisons of complementary feeding indicators and associated factors in children aged 6–23 months across five south Asian countries. Mater Child Nutr.

[37] Na M (2018). Stagnating trends in complementary feeding practices in Bangladesh: an analysis of national surveys from 2004-2014. Matern Child Nutr.

[38] Anin SK (2020). Association between Infant and Young Child Feeding (IYCF) Indicators and the Nutritional Status of Children (6–23 Months) in Northern Ghana. Nutr Res Pract.

[39] Onyango AW (2003). Dietary diversity, child nutrition, and health in contemporary African communities. Comp Biochem Physiol A Mol Integr Physiol.

[40] Guirindola MO (2018). Determinants of meeting the minimum acceptable diet among Filipino children aged 6-23 months. Philipp J Sci.

[41] Issaka AI (2015). Determinants of suboptimal complementary feeding practices among children aged 6–23 months in seven francophone west a African countries. Matern Child Nutr.

[42] Ahoya B (2019). Accelerating progress for complementary feeding in Kenya: key government actions and the way forward. Matern Child Nutr.

[43] Tassew AA (2019). Factors affecting feeding 6-23 months age children according to minimum acceptable diet in Ethiopia: a multilevel analysis of the Ethiopian demographic health survey. PLoS One.

[44] CentralStatisticalAgency, A.A., Ethiopia Demographic and health survey. 2016.

[45] USAID (2015). Guinea Nutrition Assessment.

[46] Singh S, Srivastava S, Chauhan S (2020). Inequality in child undernutrition among urban population in India: a decomposition analysis. BMC Public Health.

[47] Srivastava S, Kumar Do S (2021). Socio-economic inequality exist in micro-nutrients supplementation among children aged 6–59 months in India? Evidence from National Family Health Survey 2005–06 and 2015–16. BMC Public Health.

[48] Ogbo FA (2015). Trends in complementary feeding indicators in Nigeria, 2003–2013. BMJ Open.

[49] Kambale RM (2021). Minimum acceptable diet among children aged 6–23 months in south Kivu, Democratic Republic of Congo: a community-based cross-sectional study. BMC Pediatr.

[50] Kumar A, Kumari D, Singh A (2015). Increasing socioeconomic inequality in childhood undernutrition in urban India: trends between 1992–93, 1998–99 and 2005–06. Health Policy Plan.

[51] Reed BA, Habicht J-P, Niameogo C (1996). The effects of maternal education on child nutritional status depend on socio-environmental conditions. Int J Epidemiol.

[52] Leroy JL (2014). Maternal education mitigates the negative effects of higher income on the double burden of child stunting and maternal overweight in rural Mexico. J Nutr.

[53] Yiengprugsawan V (2007). Measuring and decomposing inequity in self-reported morbidity and self-assessed health in Thailand. Int J Equity Health.

[54] Smith LC, Ruel MT, Ndiaye A (2005). Why is child malnutrition lower in urban than in rural areas? Evidence from 36 developing countries. World Dev.

