# Supplementary figures and images for: Does socioeconomic inequality exist in minimum acceptable diet intake among children aged 6–23 months in sub-Saharan Africa? Evidence from 33 sub-Saharan African countries’ demographic and health surveys from 2010 to 2020
Source: BMC Nutr. 2022 Apr 7;8:30. doi: 10.1186/s40795-022-00521-y (PMC8991825; doi:10.1186/s40795-022-00521-y)

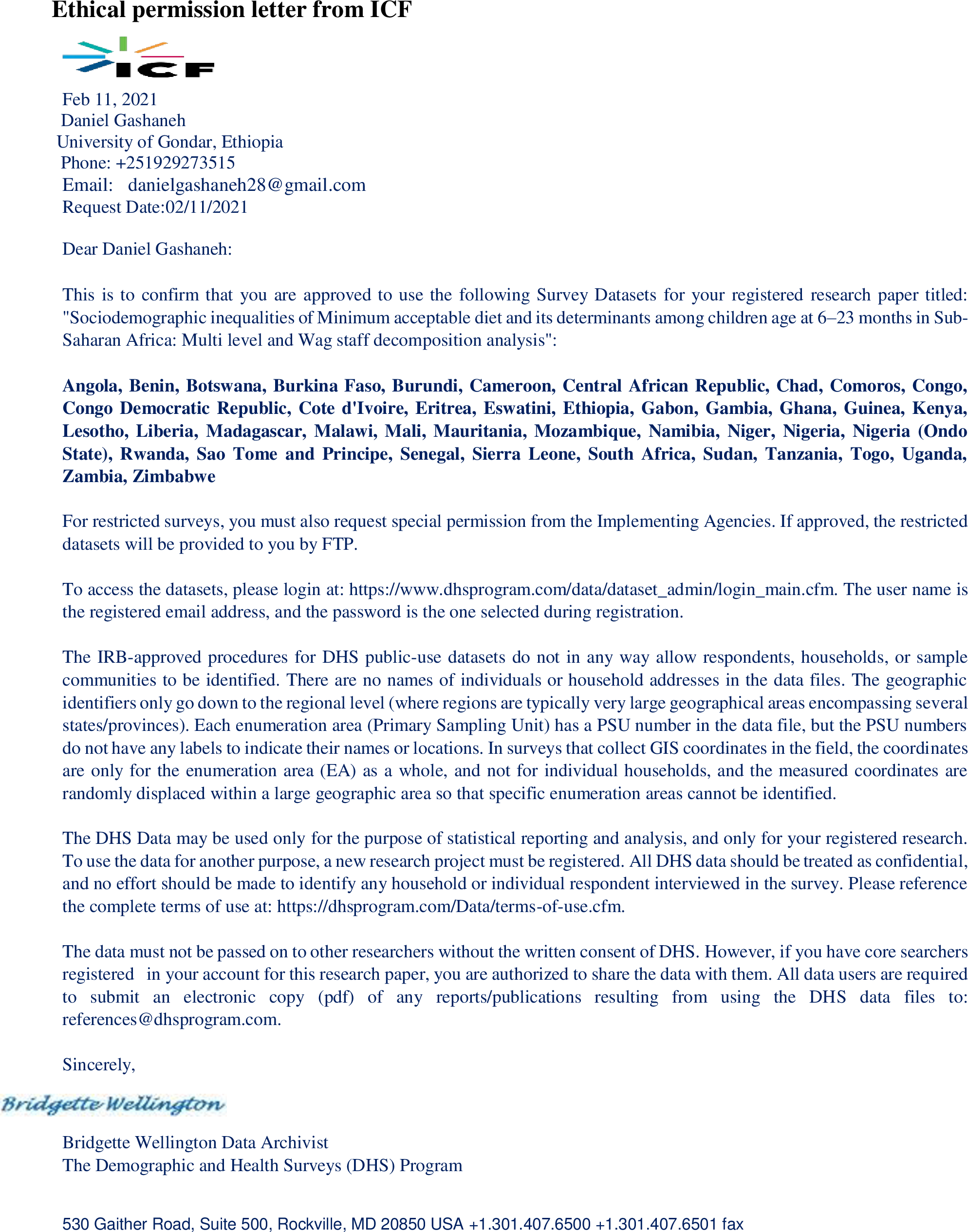


**Suplementary file**; Ethical permission letter from ICF.

Supplement: Supplementary file 1 — Additional file 1. Ethical clearance from the International Review Board of Demographic and Health Surveys (DHS) program data archivists. [file 40795_2022_521_MOESM1_ESM.docx]
